# Supplementary material for: Surface Microbiome Profiling of Dental Elevators Using Third‐Generation Sequencing: Implications for Infection Control in Dental Practice
Source: Microbiologyopen. 2025 Dec 8;14(6):e70178. doi: 10.1002/mbo3.70178 (PMC12685768; doi:10.1002/mbo3.70178)
Supplement: Supplementary file 1 — customer_backup. [file MBO3-14-e70178-s001.zip › customer_backup/customer_backup/taxa_summary/krona/groups/treat/M16.Krona.html]

Javascript must be enabled to view this page.

members
magnitude
magnitudeUnassigned

M16.krona

61003

61003

0

0

0

0

0

0

0

0

0

0

0

0

0

0

0

0

0

0

0

0

0

0

0

0

0

0

0

0

0

0

0

0

0

0

0

0

0

0

0

0

0

0

0

0

0

0

0

0

0

0

0

0

0

0

0

0

0

0

0

0

0

0

0

0

0

0

0

0

0

0

0

0

0

0

0

0

0

0

0

0

0

0

0

0

0

0

0

0

0

0

0

0

0

0

0

0

0

0

0

0

0

0

0

0

0

0

0

0

0

0

0

0

0

0

0

0

0

0

1596

1440

1440

1422

1422

1422

18

0

0

0

18

1

0

17

0

0

0

0

0

0

0

0

0

0

0

0

0

0

0

0

0

0

0

0

0

0

0

0

0

0

0

0

0

0

0

0

0

0

0

0

0

0

0

0

0

0

0

0

0

0

0

0

0

0

0

0

0

156

156

0

0

0

0

0

0

0

0

0

0

0

0

0

0

0

0

0

0

0

0

0

0

0

0

156

0

0

0

0

0

156

156

0

0

0

0

0

0

0

0

0

0

0

0

0

0

0

0

0

0

0

0

0

0

0

0

0

0

0

0

0

0

0

0

0

0

0

0

0

0

0

0

0

0

0

0

0

0

0

0

0

0

0

0

0

0

0

0

0

0

0

0

0

0

0

0

0

0

0

0

0

0

0

0

0

0

0

0

0

0

0

0

0

0

0

0

0

0

0

0

0

0

0

0

0

0

0

0

0

0

0

0

0

0

0

0

0

0

0

12

0

0

0

0

0

0

0

0

0

0

0

0

0

0

0

0

0

0

0

0

0

0

0

0

12

0

0

0

0

0

0

0

0

0

0

0

0

0

0

0

0

0

0

0

0

0

0

0

0

12

0

0

0

0

0

0

0

0

0

0

0

0

0

0

0

0

0

0

0

0

0

0

0

0

12

12

0

12

0

0

0

0

0

0

0

0

0

0

0

0

0

0

0

0

0

0

2446

24

0

0

0

0

0

0

0

0

0

0

0

0

0

0

0

0

0

0

0

0

0

0

0

0

0

0

0

0

0

0

0

0

0

0

0

0

0

0

0

0

0

0

0

0

0

0

0

0

0

0

0

0

0

0

0

0

0

0

0

0

0

0

0

0

0

0

0

0

0

0

0

0

0

0

0

0

0

0

0

0

0

0

0

0

0

0

0

0

0

0

0

0

0

0

0

0

0

0

0

0

0

0

0

0

0

0

0

0

0

0

0

0

0

0

0

0

0

0

0

0

0

0

0

0

0

0

0

0

0

0

0

0

0

0

0

0

0

0

0

0

0

0

0

0

0

0

0

0

0

0

0

0

0

0

10

0

0

0

0

0

0

0

0

0

0

0

0

0

0

0

0

0

0

0

0

0

0

0

0

0

0

0

0

0

0

0

0

0

0

0

0

0

0

0

0

0

0

0

0

0

0

0

0

0

0

0

0

0

0

0

0

0

0

0

0

0

0

0

0

0

0

0

0

0

0

0

0

0

0

0

0

0

0

0

0

0

0

0

0

0

0

0

0

0

0

0

0

0

0

0

0

0

0

0

0

0

0

0

0

0

0

0

0

0

0

0

0

0

0

0

0

0

0

0

1

1

1

0

0

0

0

0

0

0

0

0

0

0

0

0

0

0

1

0

0

0

1

1

0

7

1

0

1

0

0

5

5

0

0

0

0

0

1

1

0

0

0

0

0

0

0

0

0

0

0

1

0

0

0

0

0

0

0

0

0

0

0

0

0

0

0

0

0

0

0

0

0

0

0

0

0

0

0

0

0

0

0

0

0

0

0

0

0

0

0

0

1

1

0

0

0

0

0

0

14

0

0

0

0

0

14

0

0

0

0

0

0

0

0

0

0

0

0

0

14

14

0

0

0

0

0

0

0

0

0

0

0

0

0

0

0

0

0

0

0

0

0

0

0

0

0

0

0

0

0

0

0

0

0

0

0

0

0

0

0

0

0

0

0

0

0

0

0

0

0

0

0

0

0

0

0

0

0

0

0

0

0

0

0

0

0

0

0

0

0

0

0

0

0

0

0

0

0

0

0

0

0

0

0

0

0

0

0

0

0

0

0

0

0

0

0

0

0

0

0

0

0

0

0

0

0

0

0

0

0

0

0

0

0

0

0

0

0

0

0

0

0

0

0

0

0

0

0

0

0

0

0

0

0

0

0

0

0

0

0

0

0

0

0

0

0

0

0

0

0

0

0

0

0

0

0

0

0

0

0

0

0

0

0

0

0

0

0

0

0

0

0

0

0

2422

0

0

0

0

0

0

0

0

0

0

0

0

0

0

0

0

0

0

0

2013

356

0

0

356

356

0

0

0

0

0

0

0

0

0

0

0

0

0

0

0

0

0

0

0

0

0

0

0

0

0

0

0

0

0

0

7

0

0

0

0

0

0

0

0

0

0

0

7

0

0

0

0

0

0

0

0

0

6

1

0

0

0

0

0

0

0

0

0

0

0

0

0

0

0

0

0

0

0

0

0

0

0

0

984

0

0

0

0

984

984

0

0

0

0

0

0

0

0

0

0

276

45

45

0

0

68

53

15

163

0

49

114

0

0

0

368

0

0

0

0

0

0

0

0

0

0

0

0

0

0

0

0

0

0

0

0

0

104

0

104

22

22

0

0

0

13

0

0

13

0

0

0

0

0

229

229

0

0

22

0

0

22

17

5

0

0

0

0

0

0

0

0

0

0

0

0

0

0

0

0

0

0

0

0

0

0

0

0

0

0

0

0

168

168

168

55

113

0

0

0

0

0

0

0

0

0

0

0

0

0

0

0

0

0

0

0

35

0

0

0

17

1

1

0

0

0

0

0

0

0

16

0

0

0

16

0

0

0

0

0

0

0

0

0

0

0

0

0

0

0

0

0

0

0

0

0

18

18

0

0

18

0

0

0

0

0

0

0

0

0

0

0

0

0

0

0

0

0

0

0

0

0

0

0

0

0

0

0

0

0

0

0

0

0

0

0

0

0

0

0

0

0

0

0

0

0

0

0

0

0

0

0

0

0

0

0

0

0

0

0

206

0

0

0

0

0

0

0

0

0

0

0

0

0

0

0

0

0

2

0

0

0

0

2

2

0

0

0

0

0

0

0

0

0

0

0

0

0

0

0

204

9

9

0

0

1

1

0

0

179

0

126

26

27

15

15

0

0

0

0

0

0

0

0

0

0

0

0

0

0

0

0

0

0

0

0

0

0

0

0

0

0

0

0

0

0

0

0

0

0

0

0

0

0

0

0

0

0

0

0

0

0

0

0

0

0

0

0

0

0

0

0

0

0

0

0

0

0

0

0

0

0

0

0

0

0

0

0

0

0

0

0

0

0

0

0

0

0

0

0

0

0

0

0

0

0

0

0

0

0

0

0

0

0

0

0

0

0

0

0

0

0

0

0

0

0

0

0

0

0

0

0

0

0

0

0

0

0

0

0

0

0

0

0

0

0

0

0

0

0

0

0

0

0

0

0

0

0

0

0

0

0

0

0

0

0

0

0

0

0

0

0

0

0

0

0

0

0

0

0

0

0

0

0

0

0

0

0

0

0

0

0

0

0

0

0

0

0

0

0

0

0

0

0

0

0

0

0

0

0

0

0

0

0

0

0

0

0

0

0

0

0

0

0

0

0

0

0

0

0

0

0

0

0

0

0

0

0

0

0

0

0

0

0

0

0

0

0

0

0

0

0

0

0

0

0

0

0

0

0

0

0

0

0

0

0

0

0

0

0

0

0

0

0

0

0

0

0

0

0

0

0

0

0

0

0

0

0

0

0

0

0

11444

0

0

0

0

0

11444

0

0

0

0

0

0

0

0

0

0

0

0

0

0

0

0

0

0

0

0

0

0

0

0

0

0

0

0

0

0

0

0

0

0

0

0

0

0

0

0

0

0

0

0

0

0

0

0

0

0

0

0

0

0

0

0

0

0

0

0

0

0

0

0

0

0

0

694

0

0

0

694

0

0

0

0

0

0

0

0

0

0

0

0

0

0

0

0

0

0

0

0

0

694

227

0

433

23

0

11

0

0

0

0

0

0

0

0

0

0

0

0

0

0

0

0

0

0

0

0

0

0

0

0

0

0

0

0

0

0

0

0

0

0

0

10723

0

0

0

0

0

0

0

0

0

0

0

296

0

0

0

0

4

4

292

292

134

15

15

0

0

0

116

106

0

0

10

0

0

0

3

3

0

0

0

0

0

0

0

0

0

333

333

333

4

0

0

4

4

0

0

0

0

0

0

0

0

0

0

0

0

244

244

0

2

0

0

31

18

69

0

0

0

0

90

28

6

0

0

0

1659

0

0

1581

1183

398

78

58

0

0

20

0

0

2208

66

0

66

0

0

0

113

0

0

0

113

0

72

72

262

262

30

30

1267

3

460

2

19

27

0

25

0

0

0

11

92

105

36

0

259

228

398

0

1

145

2

35

19

5

17

0

0

54

0

0

50

5

65

0

0

0

0

0

0

5586

5586

0

0

21

5563

2

0

0

0

0

259

259

259

27

0

0

0

0

0

0

0

0

0

0

0

0

27

0

0

0

0

0

0

27

0

27

0

0

0

0

0

0

0

0

0

0

0

0

0

0

0

0

0

0

0

0

0

0

0

0

0

0

0

0

0

0

0

0

0

0

0

0

0

0

0

0

0

0

0

0

0

0

0

0

0

0

0

0

0

0

0

0

0

0

0

0

0

0

0

0

0

0

0

0

0

0

0

0

0

0

0

0

0

0

0

0

0

0

0

0

0

0

0

0

0

0

0

0

0

0

0

0

0

0

0

0

0

0

0

0

0

0

0

0

0

0

0

0

0

0

0

0

0

0

0

0

0

0

0

0

0

0

0

0

0

0

0

0

0

0

0

0

0

0

0

0

0

0

0

0

0

0

0

0

0

0

0

0

0

0

0

0

0

0

0

0

0

0

0

0

0

0

0

0

0

0

0

0

0

0

0

0

0

0

0

0

0

710

86

86

0

0

0

85

14

14

0

0

0

71

0

71

0

0

1

0

0

1

0

1

0

0

0

0

0

0

0

0

0

0

0

0

0

0

0

0

0

0

0

0

0

0

0

0

0

0

0

0

0

0

0

0

0

0

0

0

0

0

0

0

0

0

0

0

0

0

0

0

0

0

0

0

0

0

0

0

0

0

0

0

0

0

0

0

0

0

0

0

0

0

0

0

0

0

0

0

0

0

0

0

0

0

0

0

0

0

0

0

0

0

0

0

0

0

0

0

0

0

0

0

0

0

0

0

0

0

0

0

0

0

0

0

0

0

0

0

0

0

0

0

0

0

0

0

0

0

3

2

0

0

0

0

0

0

0

0

0

0

0

0

0

0

0

0

0

0

0

0

0

0

0

0

0

0

0

2

0

0

0

0

2

2

0

0

0

0

1

0

0

0

0

0

1

0

0

0

0

1

1

621

0

0

0

0

0

0

0

0

0

0

0

0

0

0

0

0

0

0

0

0

0

0

0

0

0

0

0

0

0

0

0

0

0

0

0

0

0

0

0

0

0

0

0

0

0

0

0

0

0

0

0

0

0

0

0

0

0

0

0

0

0

0

73

73

73

0

55

3

15

0

0

0

0

0

0

0

0

0

0

0

0

0

0

0

0

0

0

12

12

9

9

0

3

0

3

0

0

0

0

0

0

0

0

0

0

0

0

0

0

0

0

0

0

0

0

0

0

0

0

0

0

0

0

0

0

0

0

0

0

0

0

0

0

0

0

0

0

0

0

0

0

0

0

0

0

0

0

512

512

49

44

0

5

463

9

11

181

74

11

30

35

0

0

0

5

99

8

0

0

0

0

0

0

0

0

0

0

0

0

0

0

0

0

0

0

0

7

5

0

0

0

0

1

1

0

0

0

0

0

0

0

0

0

0

4

0

4

0

0

0

2

1

1

0

0

0

0

1

0

0

0

0

0

0

1

0

0

0

0

0

0

0

0

0

0

0

0

0

0

0

0

0

0

0

0

0

0

0

0

0

0

0

0

0

0

0

0

0

0

0

0

0

0

0

0

0

0

0

0

0

0

0

0

0

0

0

0

0

0

17

17

1

1

16

16

0

0

0

0

0

0

0

0

0

0

0

0

0

0

0

0

0

0

0

0

0

0

0

0

0

0

0

0

0

0

0

0

0

0

0

0

0

0

0

0

0

0

0

0

0

0

0

0

0

0

0

0

0

0

0

0

0

0

0

0

0

0

0

0

0

0

0

0

0

0

0

0

0

0

0

0

0

0

0

0

0

0

0

0

0

0

0

0

0

0

0

0

0

0

0

0

0

0

0

0

0

331

331

331

331

0

0

331

0

0

0

5

0

43

0

6

13

144

115

4

0

0

0

0

0

1

0

0

0

0

0

0

0

0

0

0

0

0

0

0

0

0

0

0

0

0

0

0

0

0

0

14046

14046

14046

13632

0

0

0

0

0

0

13632

13369

263

414

414

54

0

56

65

1

12

209

0

17

3972

3972

3972

0

0

0

0

0

0

0

3972

3972

458

3514

0

0

0

0

0

0

0

0

0

0

0

0

0

0

0

0

0

0

0

0

0

0

0

0

0

0

0

0

0

0

0

0

0

0

0

0

0

0

0

0

0

0

0

0

0

0

1

0

0

0

0

0

0

0

0

0

0

0

0

0

0

0

0

0

0

0

0

0

0

0

0

0

0

0

0

0

0

0

0

0

0

0

0

0

0

0

0

0

0

0

0

0

0

0

0

0

0

0

0

0

0

0

0

0

0

0

0

0

0

0

0

0

0

0

0

0

0

0

0

0

0

0

0

0

0

0

0

0

0

0

0

0

0

0

0

0

0

0

1

1

0

0

0

0

0

0

1

1

1

0

0

0

0

7247

0

0

0

0

0

0

0

0

0

0

0

0

0

0

0

0

0

0

0

0

0

0

0

0

0

0

0

0

0

0

0

0

0

0

0

0

0

0

0

0

0

0

0

0

4

4

4

4

4

0

0

0

0

0

0

0

0

0

0

0

0

0

0

0

0

0

0

0

7243

7243

0

0

0

0

0

0

2303

2303

2303

0

0

0

0

0

0

0

0

4940

67

67

41

41

0

17

17

0

0

1758

1758

3057

3057

0

0

0

0

0

0

0

0

0

0

0

0

0

0

0

0

0

0

0

0

0

1793

1793

1793

1793

0

0

1709

324

1332

53

38

38

46

46

0

0

0

0

0

0

0

0

0

0

0

0

0

0

0

0

0

0

0

0

0

0

0

0

0

0

0

0

0

0

0

0

0

0

0

0

0

0

0

0

0

0

0

0

0

0

0

0

0

0

0

0

0

0

0

0

0

0

0

0

0

0

0

0

0

0

0

0

0

0

0

0

0

0

0

0

0

0

17400

7867

28

28

15

0

15

0

0

13

0

0

13

0

0

0

169

169

158

158

11

11

286

43

43

43

0

0

0

243

0

0

243

243

0

0

0

0

0

0

1112

976

0

0

0

0

116

76

0

7

33

4

0

4

0

0

0

2

2

0

0

0

0

0

0

0

0

0

0

0

0

0

0

0

0

0

0

0

0

0

38

37

0

1

0

0

20

0

11

0

0

0

0

0

0

0

0

8

0

0

0

0

0

0

0

0

1

14

14

14

14

44

28

1

0

15

1

0

1

0

65

0

0

63

0

0

2

12

0

0

0

1

11

0

0

0

0

0

0

67

0

0

0

66

1

6

6

13

13

108

108

107

107

0

0

0

0

0

0

38

38

0

0

0

0

3

0

1

2

0

0

0

0

0

3

3

0

0

0

0

0

0

0

0

0

0

2

0

2

0

0

0

69

55

8

0

1

0

5

0

0

0

0

0

0

230

80

150

0

0

0

136

136

0

136

5819

1674

437

437

101

0

29

72

36

36

0

0

0

437

423

14

0

0

663

235

59

227

142

0

0

0

0

0

0

3699

1

1

0

0

2295

2295

1113

1113

195

195

0

0

95

95

0

446

0

0

0

0

0

0

0

0

0

121

121

325

325

18

18

0

0

0

0

18

18

0

0

0

0

0

0

0

0

69

0

0

0

50

50

50

0

0

0

0

0

0

19

19

19

11

11

11

0

11

0

0

0

0

355

278

0

0

0

0

0

0

0

0

0

0

0

0

0

0

20

0

15

0

5

0

0

0

0

0

0

0

0

0

175

1

39

0

135

0

21

7

0

14

0

0

0

0

0

0

12

12

0

0

0

0

0

0

50

0

0

0

50

17

17

17

0

0

0

17

0

0

0

17

0

0

17

0

0

0

0

0

0

0

0

0

0

0

0

0

0

43

0

0

0

0

0

0

0

23

23

0

0

0

0

0

0

20

20

0

0

0

0

0

0

0

0

0

0

0

0

0

0

0

0

0

0

0

0

0

0

0

0

0

0

0

0

0

0

0

0

0

5344

0

0

0

0

75

19

0

0

0

0

18

18

0

0

0

0

0

0

0

0

0

1

1

0

0

0

0

0

0

56

0

0

32

0

32

0

0

24

0

0

0

0

0

0

0

0

0

22

0

1

1

0

0

0

0

0

0

0

0

0

0

0

0

0

0

0

0

0

1160

98

0

0

0

0

6

6

24

0

0

24

0

0

0

68

68

0

0

0

0

0

0

0

1062

3

3

0

0

14

14

0

0

0

0

1

1

0

0

0

79

79

8

8

934

27

907

1

1

0

0

0

0

0

0

0

0

0

0

22

22

0

0

0

0

154

93

0

0

0

0

93

0

93

61

61

61

0

0

0

0

0

0

0

1

1

0

0

0

0

1

0

0

0

0

0

0

0

0

0

0

0

0

0

0

0

0

0

0

0

1

0

0

0

0

0

0

0

0

0

0

0

0

0

0

6

0

0

0

0

0

0

6

6

6

0

0

0

3

3

3

0

3

3838

0

0

0

0

20

0

0

0

0

0

0

20

20

0

0

328

321

0

0

1

0

158

0

0

1

118

0

43

0

0

7

7

0

8

8

0

8

3482

0

0

2

2

15

12

2

1

2

0

0

2

2221

1

645

1575

0

0

0

3

3

491

491

0

0

0

0

0

5

5

738

0

0

738

1

1

0

3

3

1

1

0

0

0

0

0

0

0

0

0

0

0

107

107

107

73

0

0

0

34

0

0

0

0

0

0

0

0

0

0

0

0

0

0

0

0

0

0

0

0

0

0

0

0

4189

0

0

0

0

0

0

4189

0

0

0

1780

0

0

0

0

0

0

0

0

0

1780

594

154

59

11

0

520

9

152

281

2409

113

113

0

318

318

1967

0

1219

748

0

11

11

0

0

0

0

0

0

0

0

0

5

0

0

0

0

0

0

0

0

0

0

0

0

0

0

0

0

0

0

0

0

0

0

0

0

0

0

0

0

0

0

0

0

0

0

0

0

0

0

0

0

5

0

0

0

0

5

5

0

0

0

0

0

0

5

5

0

0

0

0

0

0

0

0

0

0

0

0

0

0

0

0

0

0

0

0

0

0

0

0

0

0

0

0

0

0

0

0

0

0

0

0

0

0

0

0

0

0

0

0

0

0

0

0

0

0

0

0

0

0

0

0

0

0

0

0

0

0

0

0

0

0

0

0

0

0

0

0

0

0

0

0

0

0

0

0

0

0

0

0

0

0

0

0

0

0

0

0

0

0

0

0

0

0

0

0

0

0

0

0

0

0

0

0
